# Vitamin D insufficiency and deficiency with stages of chronic kidney disease in an Asian population

**DOI:** 10.1186/1471-2369-14-206

**Published:** 2013-10-02

**Authors:** Bancha Satirapoj, Pokkrong Limwannata, Amnart Chaiprasert, Ouppatham Supasyndh, Panbuppa Choovichian

**Affiliations:** 1 Division of Nephrology, Department of Medicine, Phramongkutklao Hospital, Bangkok, Thailand

**Keywords:** Vitamin D deficiency, Chronic kidney disease, 25-hydroxyvitamin D

## Abstract

**Background:**

Vitamin D insufficiency is associated with proteinuria and could be a risk factor for end-stage renal disease (ESRD). However, few studies have examined the significance of vitamin D insufficiency as a contributing factor for the development of ESRD in the Asian chronic kidney disease (CKD) population.

**Methods:**

Authors examined the relationship between vitamin D status and the staging of CKD using data from an outpatient clinic-based screening in 2,895 Thai CKD patients. Serum levels of 25-hydroxyvitamin D were analyzed according to CKD stages. Vitamin D deficiency and insufficiency were defined as a serum 25-hydroxyvitamin D concentration < 10 ng/mL and 10–30 ng/mL, respectively.

**Results:**

The mean (SD) 25-hydroxyvitamin D levels were significantly lower according to severity of renal impairment (CKD stage 3a: 27.84±14.03 ng/mL, CKD stage 3b: 25.86±11.14 ng/mL, CKD stage 4: 24.09±11.65 and CKD stage 5: 20.82±9.86 ng/mL, p<0.001). The prevalence of vitamin D deficiency/insufficiency was from CKD stage 3a, 3b, 4 to 5, 66.6%, 70.9%, 74.6%, and 84.7% (p<0.001). The odds ratio (95% CI) of vitamin D insufficiency/deficiency (serum 25-hydroxyvitamin D ≤ 30 ng/mL) and vitamin D deficiency (serum 25-hydroxyvitamin D < 10 ng/mL) for developing ESRD, after adjustment for age, gender, hemoglobin, serum albumin, calcium, phosphate and alkaline phosphatase were 2.19 (95% CI 1.07 to 4.48) and 16.76 (95% CI 4.89 to 57.49), respectively.

**Conclusion:**

This study demonstrates that 25-hydroxyvitamin D insufficiency and deficiency are more common and associated with the level of kidney function in the Thai CKD population especially advanced stage of CKD.

## Background

Presence of vitamin D deficiency, both in the general population and in patients with chronic kidney disease (CKD), is based mainly on the effects of vitamin D on calcium homeostasis and bone health. Bone disorders, mineral abnormalities and vascular calcification in individuals with moderate to advanced CKD seem to be related to a progressive deficiency of active vitamin D and worsening secondary hyperparathyroidism. Serum levels of 25-hydroxyvitamin D are also inversely associated with serum PTH level both in patients with CKD [1] and in those without this disease [2]. Serum 25-hydroxyvitamin D is also an inverse predictor of disease progression and death in patients with CKD [3,4].

A high prevalence of mineral metabolite abnormalities and vitamin D insufficiency or deficiency occurs in a large number of US adults with CKD and undergoing dialysis [5,6]. Recent observations have indicated that serum 25-hydroxyvitamin D is significantly lower in participants with a severe decrease in estimated glomerular filtration rate (GFR) compared with those with normal kidney function [7]. Therefore, the recent Kidney Disease Outcomes Quality Initiative (KDOQI) Clinical Practice Guidelines for Bone Mineral Metabolism and Disease in Chronic Kidney Disease recommend the measurements of 25-hydroxyvitamin D levels in patients with CKD not yet on dialysis.

Currently, clinical evidence supporting a strong link between vitamin D insufficiency or deficiency and the risk of CKD, CVD and infectious diseases both in the general population and in patients with CKD, is rapidly accumulating [8-11]. Previous reports come from small clinic-based samples and may not represent the true association between vitamin D status and kidney function in the CKD population [12,13]. In addition, a higher serum vitamin D level is expected in inhabitants of the tropics vis-a-vis inhabitants of nontropical regions, due to greater sun exposure and increased production of vitamin D. Thai patients with CKD are more likely to have high sun exposure; however, no clinical study in the Thai CKD population has been performed to support this assertion. Therefore, we determined the relationship between hypovitaminosis D and CKD stages in this population.

## Methods

A cross-sectional study was carried out in 2,895 CKD patients who followed up at the outpatient clinic of Phramongkutklao Hospital between January 1, 2010 and December 31, 2012 and had their 25-hydroxyvitamin D level assessed. All subjects did not use vitamin D supplement before being enrolled in the study. A detailed medical history was collected by reviewing medical records of the hospitals of all patients. This study was approved by our hospital Human Research Ethics Committee. All subjects gave written informed consent. For the purposes of the present study, we excluded participants with estimated GFR > 60 mL/min/1.73 m^2^ or those missing serum creatinine information.

All biochemical analyses of blood samples were conducted at the Phramongkutklao Hospital Laboratory. Serum creatinine was analyzed using the enzymatic method, calibrated to be traceable to isotope dilution mass spectrometry. An estimate of the GFR was obtained by the 2009 CKD-EPI creatinine equation [14]. Irrespective of the presence or absence of proteinuria, CKD was defined as a GFR of < 60 mL/min per 1.73 m^2^. The CKD subjects were categorized by the KDIGO Clinical Practice Guidelines for Chronic Kidney Disease in four stages: CKD stage 3a (45–59 mL/min/1.73 m^2^), CKD stage 3b (30–44 mL/min/1.73 m^2^), CKD stage 4 (15–29 mL/min/1.73 m^2^) and CKD stage 5 (<15 mL/min/1.73 m^2^).

Serum 25-hydroxyvitamin D concentrations, as a reliable measure of overall vitamin D status, were measured by electrochemiluminescence immunoassay (ECLIA) on a Roche Elecsys 10100/201 system (Roche Diagnosis Elecsys); intra- and interassay coefficients of variation were below 5% and 9%, respectively. A 25-hydroxyvitamin D deficiency was defined as having levels less than 10 ng/mL, and insufficiency, as having levels of 10 to 30 ng/mL.

### Statistical analysis

Descriptive data were examined for all variables. For continuous variables, results are presented as mean±SD. Statistical differences in variables were compared using one-way analysis of variance (ANOVA) and unpaired Student’s t-test for normally distributed variables and Kruskal-Wallis Test for non-normally distributed variables. Categorical variables were recorded as frequency counts, and intergroup comparisons were analyzed by chi-squared test. Associations between vitamin D status and CKD stage 5 were analyzed by multivariate logistic regression analysis [Odds ratio with 95% confidence intervals (CI)] and the multivariate analyses were conducted after including variables such as age, gender, hemoglobin, serum albumin, calcium, phosphate and alkaline phosphatase. Statistical significance was accepted if P<0.05. Data analysis was performed using SPSS for Windows, version 12.0 (SPSS, Chicago, IL, USA).

## Results

Subjects with CKD and estimated GFR of 34.19 ± 17.39 mL/min/1.73 m^2^ were screened for 25-hydroxyvitamin D levels. The participants were all Thais, 52.4%, male, 38.6%, had type 2 diabetes and 64.4% had hypertension as a comorbid disease. Table 1 shows the 2,895 subjects grouped to investigate for differences in clinical and laboratory characteristics according to CKD status. The values included age, gender, GFR, hemoglobin, serum albumin, calcium, phosphorus, alkaline phosphatase and intact-PTH differring among the CKD stages. Notably, levels of 25-hydroxyvitamin D were significantly lower according to severity of renal impairment (CKD stage 3a: 27.84±14.03 ng/mL, CKD stage 3b: 25.86±11.14 ng/mL, CKD stage 4: 24.09±11.65 and CKD stage 5: 20.82±9.86 ng/mL, p<0.001).

**Table 1:** Baseline characteristics of patients by CKD category

	**GFR <15 mL/min/1.73 m**^**2**^	**GFR 15–29 mL/min/1.73 m**^**2**^	**GFR 30–44 mL/min/1.73 m**^**2**^	**GFR 45–59 mL/min/1.73 m**^**2**^
	**(N=581)**	**(N=514)**	**(N=838)**	**(N=962)**
Age (yr)	63.34±16.89^*^	71.29±12.77^**^	72.61±11.49^*^	68.83±12.2
Male (n, %)	286 (49.2%)	258 (50.2%)	479 (57.2%)^**^	493 (51.2%)
GFR (mL/min/1.73 m^2^)	7.64±3.34^*^	22.71±4.21^*^	38.17±4.3^*^	52.9±4.21
BUN (mg/dL)	49.27±18.25^*^	34.98±11.36^*^	23.39±6.75^*^	17.96±5.12
Serum creatinine (mg/dL)	7.34±3.27^*^	2.49±0.55^*^	1.6±0.25^*^	1.23±0.19
Hemoglobin (g/dL)	10.82±1.71^*^	11.07±1.57^*^	12±1.7^*^	12.56±1.63
Serum albumin (g/dL)	3.99±0.55^*^	4.08±0.49^*^	4.25±0.39^**^	4.33±0.42
Serum calcium (mg/dL)	9.3±1^*^	9.37±0.63^*^	9.51±0.64	9.56±0.56
Serum phosphorus (mg/dL)	4.53±1.43^*^	3.66±0.56^*^	3.46±0.56	3.37±0.56
Serum 25-hydroxyvitamin D (ng/mL)	20.82±9.86^*^	24.09±11.65^*^	25.86±11.14^**^	27.84±14.03
Serum alkaline phosphatase (U/L)	168.39±329.4^*^	91.33±50.15	78.11±57.81	71.82±32.15
Serum Intact-PTH (pg/mL) (interquartile ranges)	211.5^*^ (102.2, 411.1)	83.5^*^ (54.3, 124.4)	59.9^*b*^ (42.5, 87.7)	51.8 (38.4, 77.1)

Data are mean ± SD and median with interquartile ranges; ^*^P<0.01; ^**^P<0.05 versus GFR 45–59 mL/min/1.73 m^2^.

**Figure 1:**
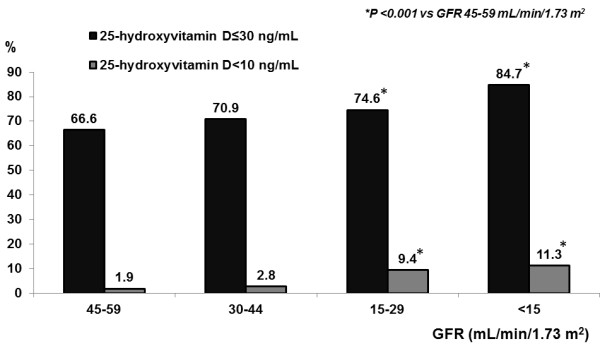
**Prevalence of vitamin D insufficiency and deficiency by CKD category.** Data are percentage; *P <0.001 versus GFR 45–59 mL/min/1.73 m^2^.

Of the 2,895 CKD patients, 72.7% had a diagnosis of 25-hydroxyvitamin D ≤ 30 ng/mL and 5.3% of 25-hydroxyvitamin D deficiency defined as less than 10 ng/mL. As shown in Figure 1, significant differences were observed in stratified patients with 25-hydroxyvitamin D ≤ 30 ng/mL according to the CKD stages (CKD stage 3a; 66.6%, CKD stage 3b; 70.9%, CKD stage 4; 74.6% and CKD stage 5; 84.7%, p<0.001). An increasing prevalence of vitamin D deficiency with increasing severity of CKD was also found (Figure 1). Males, estimated GFR, serum albumin, calcium and hemoglobin occurred significantly less among patients with levels of 25-hydroxyvitamin D ≤ 30 ng/mL. In contrast, patients with levels of 25-hydroxyvitamin D ≤ 30 ng/mL presented significantly increased serum phosphorus levels and intact-parathyroid hormone levels (Table 2).

**Table 2:** Vitamin D insufficiency and deficiency with other parameters

**Variables**	**Serum 25-hydroxyvitamin (ng/mL)**		**P-value**
	**>30 ng/mL**	**≤30 ng/mL**	
Age (yr)	70.19 ± 12.37	69.34 ± 13.59	0.271
Male (N, %)	239 (58.4%)	553 (50.7%)	0.008
GFR (mL/min/1.73 m^2^)	39.08 ± 15.83	33.22 ± 17.99	<0.001
BUN (mg/dL)	26.08 ± 13.15	30.84 ± 17.23	<0.001
Creatinine (mg/dL)	2.22 ± 2.09	2.99 ± 3.01	<0.001
Hemoglobin (g/dL)	11.84 ± 1.87	11.54 ± 1.72	0.025
Serum albumin (g/dL)	4.29 ± 0.48	4.14 ± 0.49	<0.001
Serum calcium (mg/dL)	9.53 ± 0.65	9.4 ± 0.74	0.009
Serum phosphorus (mg/dL)	3.56 ± 0.79	3.85 ± 1.08	<0.001
Serum alkaline phosphatase (U/L)	79.75 ± 41.88	97.41 ± 95.91	0.052
Serum intact-PTH (pg/mL)	64.88 (40.53, 111.8)	82.31 (46.13, 189)	0.005

Data are mean ± SD and median with interquartile ranges, P-value versus serum 25-hydroxyvitamin D >30 ng/dL.

Table 3 shows the adjusted odds ratio for advanced CKD stage in subjects categorized based on vitamin D deficiency and insufficiency. Using multivariate logistic regression analysis, an inverse association was observed between serum 25-hydroxyvitamin D ≤ 30 ng/mL and prevalent CKD stage 5 [adjusted odds ratio 2.19 (95% CI 1.07 to 4.48)] in the CKD population after adjusting for potential confounders. Additionally, the association between serum 25-hydroxyvitamin D < 10 ng/mL and prevalent CKD stage 5 [adjusted Odds ratio 16.76 (95% CI 4.89 to 57.49)] remained statistically significant in CKD participants, even after adjusting confounding factors.

**Table 3:** **Vitamin D insufficiency/deficiency associated with advanced CKD stage defined as estimated GFR less than 15 mL/min/1.73 m**^**2 **^**in the entire CKD subjects as evaluated by multiple logistic regression analysis**

**Serum 25-hydroxyvitamin D**	**Unadjusted OR**	**P-value**	**Adjusted OR**	**P-value**
	**(95%CI)**		**(95%CI)**	
≤30 ng/dL	2.15 (1.56, 2.97)	<0.001	2.19 (1.07, 4.48)	0.032
10-30 ng/dL	1.93 (1.39, 2.67)	<0.001	1.84 (0.88, 3.82)	0.104
<10 ng/dL	8.72 (5.32, 14.29)	<0.001	16.76 (4.89, 57.49)	<0.001
>30 ng/dL	Reference		Reference	

Multivariate analyses were done after including variables such as age, gender, hemoglobin, serum albumin, calcium, phosphate and alkaline phosphatase. P-value versus serum 25-hydroxyvitamin D >30 ng/dL (reference).

## Discussion

The principal finding of the present study is that vitamin D insufficiency and deficiency are widely prevalent among Thai CKD patients, similar to the general population. Regardless of the geographic location in Thailand, serum 25-hydroxyvitamin D level ≤30 ng/mL was present in 66.6-84.7% of patients with stage 3a-5 CKD. Also low vitamin D status has been reported among Asian populations with normal kidney function despite it being a tropical region [15-17]. This is the first epidemiological study in Asian tropical countries to use estimated GFR calculations to demonstrate that vitamin D insufficiency and deficiency is associated with lower estimated GFR.

Our findings complement recent observations suggesting that vitamin D deficiency is strongly associated with greater stages of CKD among adult participants. Among 14,679 US adult participants in the Third National Health and Nutrition Examination Survey (NHANES III), mean serum 25-hydroxyvitamin D level was lower in patients with stage 4–5 CKD compared with those with normal kidney function (24.6 vs. 29.3 ng/mL, *P* <0.001) [7]. Similarly, another study measured serum 25-hydroxyvitamin D levels in patients with CKD. The overall mean serum level of 25-hydroxyvitamin D was 19 ±14 ng/mL and only 29% of the 65 patients with stage 3 CKD and only 17% of 113 patients with stage 4 CKD had vitamin D insufficiency and deficiency [18]. Moreover, participants with 25-hydroxyvitamin D levels <15 ng/mL had a 2.6-fold greater incidence of ESRD than those with levels ≥15 ng/mL during a long-term follow-up [19]. In addition, few studies demonstrated that low 25-hydroxyvitamin D levels were independently associated with albuminuria in CKD and type 1 diabetes, but they did not find evidence linking low concentrations of 25-hydroxyvitamin D to early GFR loss [20,21]. Thus, current data and our finding indicate that vitamin D deficiency/insufficiency is an extremely frequent condition in patients with CKD, especially those with an estimated GFR of less than 15 mL/min/1.73 m^2^.

Our results demonstrate a graded relationship between serum 25-hydroxyvitamin D and the risk for kidney disease among subjects with CKD not undergoing dialysis. A causal relationship has yet to be proved by intervention trials using vitamin D. Several mechanisms might explain the 25-hydroxyvitamin D deficiency in the CKD population. First, almost patients with CKD have restricted protein and caloric intake, so vitamin D is relatively low [22]. Second, many CKD patients have limited outdoor physical activities with reduced exposure to sunlight [22]. Finally, greater loss of urinary vitamin D metabolites occurs in patients with overt proteinuria [23].

Increasing evidence supports that vitamin D metabolism affects the risk of CKD, although the underlying molecular mechanism of this association remains hidden. Mounting evidence from clinical studies has demonstrated an inverse relationship between circulating vitamin D levels and blood pressure and/or abnormalities of the renin angiotensin-aldosterone system (RAAS). The RAAS plays a key role in regulating blood pressure, vascular remodeling and progressive kidney damage [24]. In animal models, inhibiting 1,25(OH)(2)D synthesis led to an increase in renin expression, whereas injecting 1,25(OH)(2)D led to renin suppression [25]. Hence, 1,25(OH)(2)D is a novel negative endocrine regulator of the RAAS [26]. Moreover, replacement with pharmacologic doses of vitamin D receptor agonists in animal models of kidney disease consistently show reduced albuminuria, abrogated glomerulosclerosis, and glomerular inflammation [27,28]. Recently, a cohort study found that serum 25-hydroxyvitamin D concentration was an independent inverse predictor of disease progression and death in patients with stages 2–5 CKD [3]. Collectively, these data suggest a potential renoprotective effect of vitamin D supplementation in patients with advanced CKD.

This study has several strengths including the large number of participants, the complete nature of the dataset and the ability to adjust to multiple CKD risk factors. Despite the comprehensive nature of the dataset, limitations occurred in the study. We enrolled only Thai CKD patients in Bangkok, so caution is needed in generalizing our finding with other populations. The CKD EPI formula was not tested for accuracy in Thai population and thus may be used for relative measurements of GFR and comparisons between vitamin D deficiency and non-vitamin D deficiency. Finally, our selection of subjects might have been biased. Our participants were mainly in the tertiary care center. This might be one reason why more aging and chronically ill subjects participated.

## Conclusion

In summary, the present data indicate a high prevalence of 25-hydroxyvitamin D deficiency and insufficiency in Thai patients with moderate and severe CKD not on dialysis. Even though Thailand is in a tropical region, most patients had suboptimal levels of serum vitamin D. Especially among advanced stage CKD patients, 25-hydroxyvitamin D deficiency is strongly and independently associated with CKD. The results of our study raise an important public health issue and needs to be confirmed by large-scale and cohort studies in other populations.

## Competing interests

The authors declare that they have no competing interests.

## Authors’ contributions

SB collected the data, drafted the article, reviewed the literature and revised it critically. LP provided valuable inputs in study design, data collection and literature review. CA did statistical interpretation and provided valuable inputs in the draft. SO provided valuable inputs in data collection and literature review. CP provided literature review and revision of the draft. All authors read and approved the manuscript and met the criteria for authorship.

## Pre-publication history

The pre-publication history for this paper can be accessed here:

http://www.biomedcentral.com/1471-2369/14/206/prepub
